# The Expression of PD-1 Ligands and Their Involvement in Regulation of T Cell Functions in Acute and Chronic Woodchuck Hepatitis Virus Infection

**DOI:** 10.1371/journal.pone.0026196

**Published:** 2011-10-14

**Authors:** Ejuan Zhang, Xiaoyong Zhang, Jia Liu, Baoju Wang, Yongjun Tian, Anna D. Kosinska, Zhiyong Ma, Yang Xu, Ulf Dittmer, Michael Roggendorf, Dongliang Yang, Mengji Lu

**Affiliations:** 1 Department of Microbiology, Tongji Medical College, Huazhong University of Science and Technology, Wuhan, China; 2 Institute of Virology, University Hospital of Essen, Essen, Germany; 3 Division of Clinical Immunology, Tongji Hospital, Tongji Medical College, Huazhong University of Science and Technology, Wuhan, China; Beijing Institute of Infectious Diseases, China

## Abstract

**Background:**

The programmed cell death 1 (PD-1)/programmed death-1 ligand 1 (PD-L1) system may play a role in the negative regulation of T cell functions in hepatitis B virus (HBV) infection. Thus, it is important to study its role in the widely used animal model for HBV infection of woodchucks with woodchuck hepatitis virus (WHV).

**Methods:**

Woodchuck PD-L1 (wPD-L1) and -L2 (wPD-L2) were cloned and characterized. The levels of wPD-L1 expression in primary woodchuck hepatocytes (PWH), peripheral blood mononuclear cells (PBMCs), and liver tissue of naive and WHV-infected woodchucks were examined by real time reverse transcription (RT)-PCR and flow cytometry. Using antibodies against wPD-L1 and -L2, the effect of blocking PD-1/PD-L1/PD-L2 interaction on the proliferation and degranulation of woodchuck PBMCs was examined.

**Principal Findings:**

Both wPD-L1 and -L2 showed a high homology to their counterparts of other mammalian species and humans. WPD-L1 expression in PWH and PBMCs of naive animals was low but could be stimulated by Toll-like receptor (TLR) ligands and interferons (IFN). WPD-L1 expression in liver tissue was significantly higher than that measured in PWHs and was slightly elevated during acute and chronic WHV infection. However, wPD-1 and wPD-L1 expression on PBMCs was strongly up-regulated during acute and chronic infection. *In vitro* blockade with antibodies against wPD-L1 and -L2 partially enhanced proliferation and degranulation of PBMCs from WHV-infected woodchucks.

**Conclusions:**

Our results demonstrated that wPD-1/wPD-L1 expression in hepatocytes and PBMCs can be induced by different inflammatory stimuli and is up-regulated mainly on PBMCs during WHV infection. A blockade of the woodchuck PD-1/PD-L pathway could partially enhance T cell functions in WHV infection.

## Introduction

Hepatitis B virus (HBV) infection is a global health problem due to its high prevalence in the world. It is generally accepted that an appropriate T cell response to HBV proteins is crucial for viral elimination [1]. A spontaneous recovery from acute HBV infection is typically associated with vigorous multispecific CD4+ and CD8+ T cell responses in patients. Chronic HBV infection is characterized by the absence of specific T cells in the peripheral blood and low numbers of HBV specific CD4+ and CD8+ cells with impaired functions in the liver [1], [2].

Programmed cell death-1 (PD-1) and its ligands (PD-L1 and PD-L2) belongs to the B7/CD28 family of co-stimulatory molecules and have important functions concerning the regulation of T cell responses. Normally, PD-1 is expressed on lymphocytes at a low level but up-regulated for a short term after activation. The engagement of PD-1 to its ligands during this period leads to the negative regulation of T cells, which plays an important role in the maintenance of peripheral tolerance against immunopathological tissue damage [3]. However, the PD-1/PD-L1 system is also involved in the induction of T cell exhaustion during chronic viral infections [4], [5]. PD-1 expression on the virus-specific T cells is negatively correlated with cytokine secretion and cytotoxity and is associated with viral persistence in different chronic virus infections, such as human immunodeficiency virus, HBV, hepatitis C virus, and cytomegalovirus [6]–[8]. *In vitro* blockade of the PD-1/PD-L1 pathway using anti-PD-1 or PD-L1 antibodies could partially restore the function of exhausted cytotoxic T lymphocytes (CTLs) [9]. Moreover, an *in vivo* blockade in mice infected with lymphocytic choriomeningitis virus and HBV-transgenic mice could improve the expansion, cytokine production, and the cytolytic activity of virus-specific T cells, and lead to a rapid decrease of viral loads [10], [11]. Some hints indicate that a chronic course of HBV infection is also associated with PD-1 up-regulation on HBV-specific T cells and an enhanced PD-L1 expression in liver tissue [12]. A treatment with antibodies to PD-L1 *in vitro* improved the functions of HBV-specific T cells derived from peripheral blood and liver tissue [7], [13].

The woodchuck model is a useful animal model for studies on HBV infection. Woodchucks can be infected with woodchuck hepatitis virus (WHV), a genetically closely related virus to HBV. Like in humans, the specific T cell responses to WHV proteins are strong during the acute WHV infection and weak or absent in chronically WHV-infected woodchucks. The woodchuck model has been used to test different approaches for immunomodulation and immunotherapy of chronic HBV infection [14]–[16]. In the present study, wPD-L1 and -L2 were molecularly characterized and examined in the context of WHV infection. During acute and chronic WHV infection, the expression of both wPD-L1 and wPD-1 on PBMCs was elevated. However, there was no significant change of wPD-L1 expression in the liver tissue between naïve and infected woodchucks. A blockade of wPD-L1 *in vitro* by using specific antibodies improved the proliferation of woodchuck PBMCs and CTL functions in some but not in all animals with acute and chronic WHV infection.

## Materials and Methods

### Ethics Statement

Woodchucks were purchased from Northwest Wildlife (Ithaca, NY) and kept at the animal facilities at the University Hospital of Essen. Experiments were conducted in accordance with the Guide for the Care and Use of Laboratory Animals and were reviewed and approved by the local Animal Care and Use Committee (Animal Care Center, University of Duisburg-Essen, Essen, Germany, and the district government of Düsseldorf, Germany) with a Permit Number 8.87–50.10.34.08.281. All procedures were performed on woodchucks anesthetized with ketamine-xylazine and all efforts were made to minimize suffering.

### Study group

Chronically WHV-infected woodchucks were positive for WHV DNA, WHsAg, anti-WHc, but negative for anti-WHs. The chronic carriers were directly purchased from Northwest Wildlife. Naïve woodchucks were inoculated intravenously with10^7^ WHV genome equivalents (GE). Adult woodchucks developed mainly acute self-limiting WHV infections after inoculation and all WHV markers were cleared within 6 months. During the acute infection, the peak of viremia appeared around week 4–8 post infection. Therefore, week 8 was considered as demarcation of early and late phase of WHV infection in this study.

### Cell cultures

Baby hamster kidney (BHK) cells were maintained in Dulbecco's modified Eagle's medium supplemented with 10% fetal calf serum. Woodchuck PBMCs and PWH were prepared by Ficoll or liver perfusion and cultured as described previously [17], [18]. Experiments with PWH were performed with at least 3 different preparations.

### Stimulus and Antibodies

TLR1-9 ligands were purchased from Invivogen (USA). wIFN-α and wIFN-γ were expressed in eukaryotic cells after tranfection. WHcAg protein was expressed in E. coli and purified by chromatography through Sepharose 6 and sucrose gradient centrifugation. WHcAg-derived peptide was purchased from EMC (Tübingen, Germany). HRP labeled goat-anti-rabbit or goat-anti-mouse IgG, fluorescent labeled antibodies including goat-anti-rabbit IgG-FITC, mouse-anti-human PD-1-FITC, rat-anti-mouse PD-L1-PE, rat-anti-mouse CD3-PE, and corresponding isotype antibodies were purchased from eBioscience (USA). Rat-anti-mouse CD107a-FITC, mouse-anti-human CD4-APC, and 7-amino-actinomycin D (7aad) were purchased from BD bioscience (USA). Polyclonal rabbit anti-wPD-L1 or -L2 antibodies were prepared and tested by our group (Text S1 and S2). Total IgG purified from normal rabbits was used as a rabbit control antibody.

### DNA sequences of PD-L1 and PD-L2 of different mammalian species

The following sequences with the corresponding GenBank accession numbers were included: human PD-L1 (AY254342), woodchuck PD-L1 (EU306520), porcine PD-L1 (AY837780), mouse PD-L1 (NM_021893), cattle PD-L1 (AB510902), human PD-L2 (AF344424), woodchuck PD-L2 (EU306521), chimpanzee PD-L2 (NM_001083599), porcine PD-L2 (NM_001025220), mouse PD-L2 (NM_021396).

### Reverse transcription (RT)-PCR amplification of the coding sequences of wPD-L1 and -L2

CDNA fragments of wPD-L1 and -L2 were amplified from woodchuck liver RNA by RT-PCR with the primers list in Table S1. The specific PCR fragments (Figure S1) were cloned into pMD18-T TA vector (TAKARA) and subjected to DNA sequencing. The phylogenetic analysis of the sequence data was performed online with the software ClustalW at the website of the European Bioinformatics Institute (http://www.ebi.ac.uk). The secondary structure of protein sequences data was predicted by an online service of Swiss Institute of Bioinformatics (http://swissmodel.expasy.org). Recombinant wPD-L1 and -L2 proteins were expressed in E. coli and mammalian cells (Text S1).

### Detection of wPD-L1 expression in PWHs, woodchuck PBMCs, and liver tissue by real time RT-PCR

Woodchuck PBMCs and PWHs were treated with ligands of TLR3 (poly I∶C, 12.5 µg/ml), TLR7 (imiquimod, 10 µg/ml), and TLR9 (CpG ODN 12.5 µg/ml) by transfection with lipofectamin2000 (Invitrogen, Germany) or direct administration of ligands of TLR1/2 (Pam3Cysk4, 2 µg/ml), TLR2/6 (Pam2Cysk4, 2 µg/ml), TLR4 (lipopolysaccharid, LPS), 12.5 µg/ml), and TLR5 (flagellin, 10 µg/ml), woodchuck IFN-α and -γ (500 U/ml) for 6 h. Total RNA of cells or woodchuck liver samples were prepared for real time RT-PCR to detect the wPD-L1 mRNA as described previously [18].

### Detection of wPD-1 and -L1 expression in PWHs and woodchuck PBMCs by flow cytometry

Two commercially available, cross-reactive monoclonal antibodies, mouse-anti-human PD-1-FITC (clone J116, ebioscience, USA) and rat-anti-mouse PD-L1-PE (clone MIH5, ebioscience, USA), were used for FACS staining of PWHs and woodchuck PBMCs, mouse IgGλ, rat IgG1κ-PE were used as isotype contorls. Dead cells were excluded by live staining with 7aad (BD Pharmingen, USA). PWHs were harvested at 20 h post stimulation by incubation with trypsin-free cell dissociation buffer and stained with anti-PD-L1/7aad. Woodchuck PBMCs were stained with anti-CD3/anti-CD4/7aad/anti-PD-1 or anti-CD4/7aad/anti-PD-L1. A detailed description of flow cytometry analysis is given in Figure S2, S3A, and S3B.

### Immunofluorescence (IF) staining of wPD-L1 and -L2 proteins expressed in transient transfection and in woodchuck PBMCs

To test the recognition of wPD-L1 and -L2 by rabbit anti-woodchuck specific antibodies generated by our group (Text S2), IF staining was performed with BHK cells transient transfected with pCI-wL1 and pxf3H-wL2 by using Lipofectamin2000. Forty-eight hours after transfection, cells were fixed with 50% methanol-PBS. To detect the expression of wPD-L1 and -L2 proteins on woodchuck PBMCs, PBMCs were placed onto microscope slides and fixed. Cells were stained with rabbit anti-wPD-L1 and -L2 antibodies as described previously [19].

### Proliferation assay and CD107a degranulation assay with woodchuck PBMCs and blocking the wPD-1/PD-L pathway using specific antibodies

The PBMCs proliferation and CD107a degranulation assay were performed as described previously [18], [19]. Woodchuck PBMCs were seeded in triplicates at a density of 5×10^4^ per well for the proliferation assay and 1×10^6^ per well for the CD107a degranulation assay, and cultured in flat-bottom 96-well microtiter plates, as optimized previously. WHcAg was added to PBMCs at a final concentration of 1 µg/ml for the proliferation assay. For proliferation assay, stimulation index (SI) is calculated with the formula: (stimulated cpm - blank cpm)/(unstimulated cpm - blank cpm). To test the effect of the blockade of wPD-1/PD-L1/PD-L2 pathway, woodchuck PBMCs were incubated with different dilutions of anti-wPD-L1 (stock concentration 2.7 mg/ml), anti-wPD-L2 (stock concentration 2.5 mg/ml), anti-wPD-L1 plus -L2, or control antibody, respectively. A negative control without antibodies was included. A WHcAg-derived peptide aa 96–110, which was identified as a CD8 epitope widely recognized by a great number of woodchucks, was used for stimulation in degranulation assay. Due to the lack of available anti-CD8 antibody, woodcheuck PBMCs were stained with cross-reactive antibodies to CD3 and CD4. The CD3+CD4− lymphocytes were gated and regarded as mainly CD8+ cells, with minor fractions of CD3+CD4−CD8− cells. In this study, the CD3+CD4− T cell population were analyzed. Normally, CD3+CD4−T cell response detected by degranulation assays ranged between 0–10%, with highest values in the peak of acute WHV infection. The improvement of T-cell degranulation by the blockade of wPD-1/PD-L1/PD-L2 pathway was expressed as the fold change calculated with measured percentages of CD107+ cells in the assays with the formula: (“anti-PD-L1 plus peptide” - “peptide”)/(“control antibody plus peptide” - “peptide”). A fold change >2 was regarded as a positive result.

### Statistical analysis

The statistical analysis was automatically carried out by GraphPad software (San Diego, USA). Significant differences between the groups were determined by nonparametric Student's t-test. P<0.05 was considered as statistically significant. Data are presented as means ± standard deviation.

## Results

### Molecular cloning and characterization of wPD-L1 and -L2

**Figure 1:**
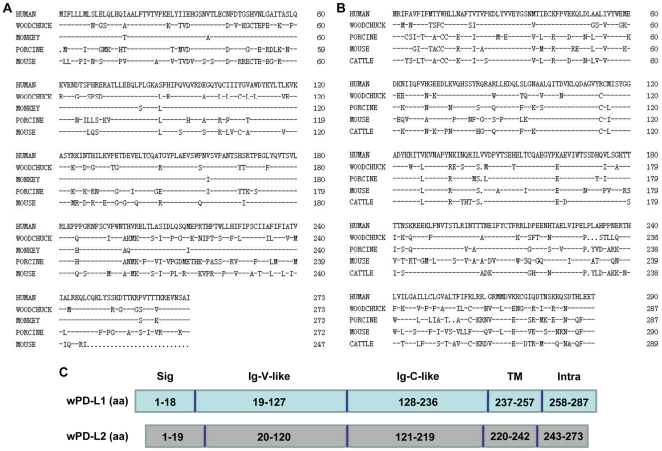
Alignment of the deduced aa sequences of PD-L1 and PD-L2 of different mammalian species. The following sequences with the corresponding GenBank accession numbers in brackets were included: human PD-L1 (AY254342), woodchuck PD-L1 (EU306520), porcine PD-L1 (AY837780), mouse PD-L1 (NM_021893), cattle PD-L1 (AB510902), human PD-L2 (AF344424), woodchuck PD-L2 (EU306521), chimpanzee PD-L2, (NM_001083599), porcine PD-L2 (NM_001025220), mouse PD-L2 (NM_021396). Alignment of wPD-L1 (A) and -L2 (B) with other mammalian species. (C) Predicted secondary structure of wPD-L1 and -L2 by online analysis. Sig, the predicted signal peptide; Ig-V-like, the immunoglobulin V-like domain; Ig-C-like, the immunoglobulin C-like domain; TM, the transmembrane region; Intra, the intracellular domain.

**Figure 2:**
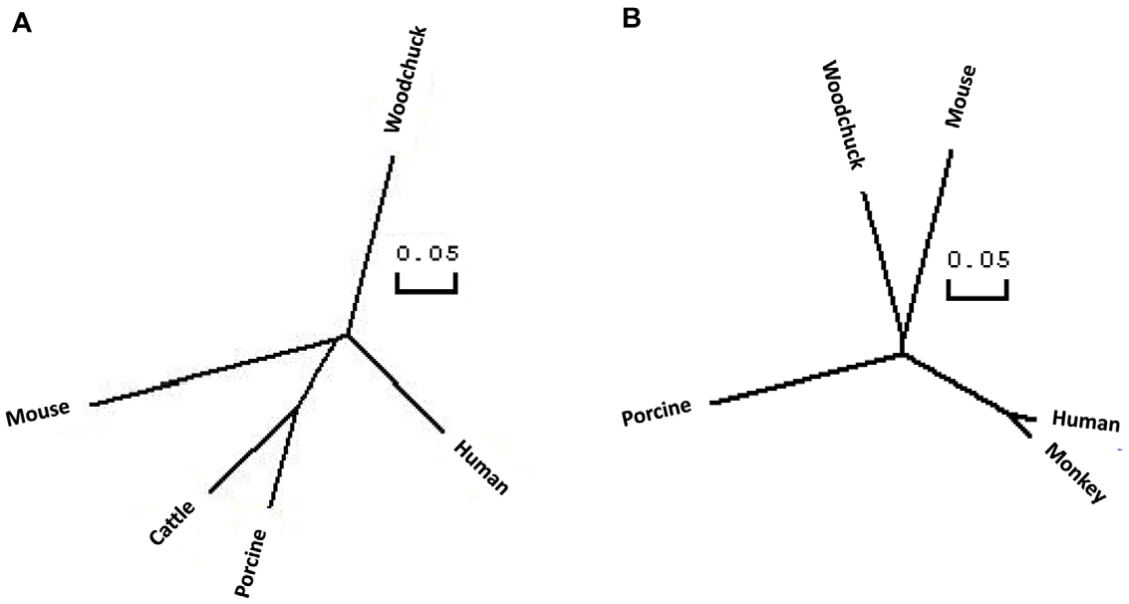
Phylogenetic trees based on the deduced aa sequences of PD-L1 and PD-L2. The phylogenetic tree of PD-L1 (A) and PD-L2 (B) of different mammalian species. The same sequences used in Fig. 1 were considered for the phylogenetic trees.

A comparison of the complete coding regions of wPD-L1 and -L2 obtained by RT-PCR revealed a high homology at the nucleotide (nt) and amino acid (aa) levels to the counterparts of mammalian species (Table S2A and S2B). The putative precursor of wPD-L1 and -L2 had a length of 287 and 274 aa residues and had a homology of 75.9% and 72.9% to their human counterparts, respectively. At the aa sequence level, both proteins showed the typical features of PD-L1 and -L2 with a signal peptide, an extracellular domain with a Ig-V-like region, a Ig-C-like region, a trans-membrane domain, and a intracellular domain (Fig. 1A, B, and C). The homology between wPD-L1 and -L2 was 31.4% for the complete coding region with a highest value of 36.2% for the extracellular domain and the lowest value of 7.4% for the intracellular domain. The phylogenetic tree shows the relative similarity of PD-L1 and -L2 of different mammalian species (Fig. 2).

### Induction of wPD-L1 expression in the PWH and PBMCs by TLR ligands and IFNs

**Figure 3:**
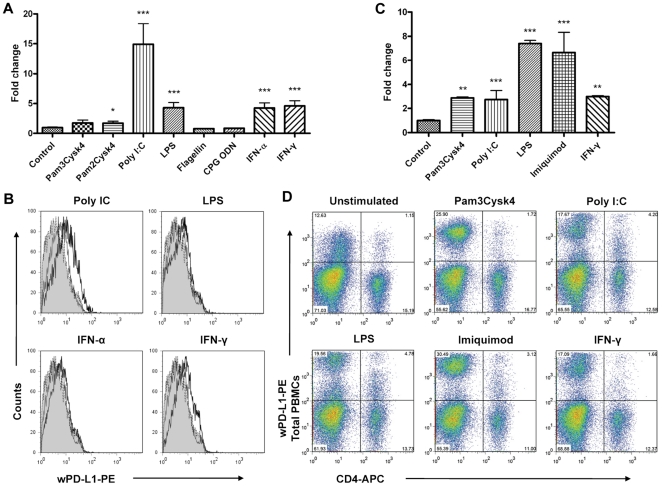
WPD-L1 expression in PWHs and PBMCs stimulated by TLR ligands. PWHs and PBMCs were stimulated with Pam3Cysk4 (2 µg/ml), Pam2Cysk4 (2 µg/ml), PolyI∶C (12.5 µg/ml), LPS (12.5 µg/ml), flagellin (10 µg/ml), imiquimod (10 µg/ml), CpG ODN (12.5 µg/ml), woodchuck IFN-α (500 U/ml), or -γ (500 U/ml). The copy numbers of wPD-L1 transcripts in PWHs (A) or PBMCs (C) were detected by real time RT-PCR after 6 h stimulation. PD-L1 protein expression on PWHs (B) or PBMCs (D) was determined by FACS staining. Shadows, isotype control antibody stained PWH; hairline, unstimulated PWH; normal lines, stimulated cells. The statistical analysis was performed for the samples treated with TLR ligands and to the unstimulated control. The statistical significance of the results was given as P values: *, p<0.05; **, p<0.005; ***, p<0.0005.

During viral infections, the expression of PD-L1 in different cell types may be stimulated by TLR ligands, or inflammatory cytokines such as IFN-α and -γ [20]–[22]. Therefore, PWHs and woodchuck PBMCs from naive or chronically WHV-infected animals were treated with TLR ligands or IFNs, and the expression of wPD-L1 was determined. PWHs were treated with the ligands for TLR1/2 (Pam3Cysk4), TLR2/6 (Pam2Cysk4), TLR3 (poly I∶C), TLR4 (LPS), TLR5 (flagellin), TLR7 (imiquimod), TLR9 (CpG ODN), woodchuck IFN-α, or -γ for 6 h or 20 h and wPD-L1 expression was detected by real time RT -PCR or flow cytometry. The base line expression of wPD-L1 in unstimulated PWHs was about 1252 copies/10^6^ wß-actin on average. Similar to human hepatoma cells, the expression of wPD-L1 was upregulated by TLR3 (14.9-fold) and TLR4 (4.3-fold) ligands, woodchuck IFN-α (4.3-fold) and -γ (4.6-fold) in PWHs (Fig. 3A, Table S3). Staining with the cross-reactive anti-human PD-L1 antibody confirmed the upregulated wPD-L1 protein expression on PWHs (Fig. 3B). In contrast, ligands for TLR2, 5, and 9 showed only a weak or no effect on the induction of wPD-L1 expression in PWHs.

In contrast, wPD-L1 expression at the level of mRNAs in woodchuck PBMCs was strongly stimulated by LPS and imiquimod (7.4- and 6.7-fold, Table S4), while Pam3Cysk4, PolyI∶C, and IFN-γ had a weaker effect on wPD-L1 expression (Fig. 3C). Analyzed by flow cytometry, a population of 10 to 30% of total PBMCs showed a low level of wPD-L1 expression after an in vitro culturing for 24 h (Fig. 3D). WPD-L1 expression was significantly upregulated by stimulation, mainly due to an increased wPD-L1 expression in the non-lymphocytes population (Figure S3C). WPD-L1 expression in lymphocytes was low without treatment. Both CD3+CD4+ and CD3+CD4− cells were responsive to the stimulation with TLR ligands and showed a modest wPD-L1 expression after treatment (Figure S3D). CD3+CD4+ cells were differently stimulated and showed a lower PD-L1 expression level compared with CD3+CD4− cells. These results were consistent with former publications that lymphocytes express PD-L1 [20], [21]. The results shown for each ligand were reproduced in 5 different animals, including 2 naive and 3 chronic carriers. However, the stimulation with Pam2Cysk4, flagellin, and CpG did not generate consistent results with PBMCs from different woodchucks, so the data were not shown.

### WPD-1 and wPD-L1 expression in acutely or chronically WHV-infected woodchucks

**Figure 4:**
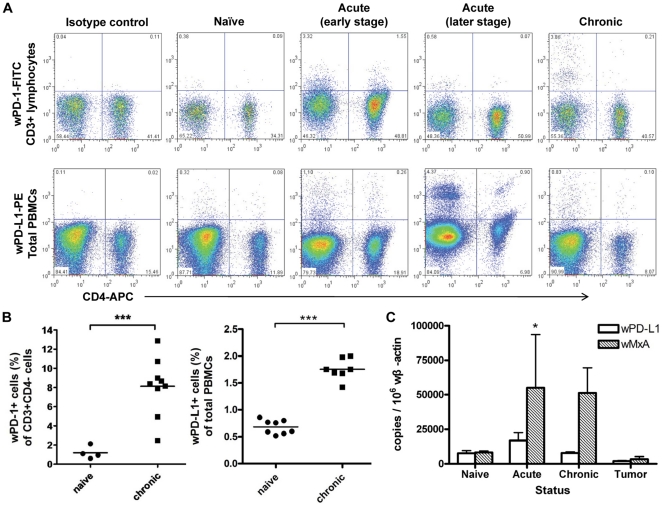
WPD-1 and wPD-L1 expression during WHV infection. Naïve, acutely and chronically WHV-infected woodchucks were examined for wPD-1 and PD-L1 expression on PBMCs and in liver tissue. (A) The expression of wPD-1 on CD3+ cells and wPD-L1 on total PBMCs from woodchucks with different status of WHV infection. (B) The expression of wPD-1 and wPD-L1 on woodchuck PBMCs was compared in naive and chronically infected woodchucks by flow cytometry analysis.. (C) Liver tissue was collected from naïve woodchucks (n = 4), woodchucks with acute (n = 5), chronic (n = 7) WHV infection, HCC tissue and peritumoural tissue (n = 3). Total mRNAs were extracted from the liver tissue and subjected to real time RT-PCR for wPD-L1 and wMxA expression. The stastical analysis was performed to compare the gene expression levels in different animals with the naïve animals as reference. The statistical significance of the results was given as P values: *, p<0.05; **, p<0.005; ***, p<0.0005.

To establish the relationship to WHV infection, wPD-1/PD-L1 expression in naïve and WHV-infected woodchucks was examined. WPD-1 and wPD-L1 expression was detected at a low level of about 1% of woodchuck PBMCs in naive woodchucks, respectively (Fig. 4A). In acutely WHV-infected woodchucks, wPD-1 expression on woodchuck PBMCs increased during the viremic phase and returned to the baseline after the viral clearance. The expression of wPD-L1 remained low during the acute phase of WHV infection and only elevated shortly at the late viremic phase (Fig. 4A). Consistent with the findings in HBV-infected patients, wPD-1 and wPD-L1 were continuously expressed at high level in chronic infected animals compared with naïve woodchucks (Fig. 4A and 4B).

It is of particular interest whether an enhanced PD-L1 expression in liver tissue may play a role in the suppression of specific T cell responses to hepadnaviral proteins. Thus, liver tissues were collected from naïve woodchucks (n = 4), woodchucks with acute (n = 5) and chronic (n = 7) WHV infection. Three available woodchuck HCC and peritumoural tissue sets were included for the quantitation of wPD-L1 transcripts. Total RNAs were prepared from liver tissue and subjected to real time RT-PCR for wβ-actin, wPD-L1, and wMxA.

The base line of wPD-L1 expression in the liver tissue of uninfected woodchucks was 7535 copies/10^6^ wβ-actin mRNAs on average and significantly higher than the level in cultured PWHs. Taken the expression level of wPD-L1 in naïve woodchucks as the base line, only a slight up-regulation of wPD-L1 was measured in acutely WHV-infected woodchucks. The expression levels of wPD-L1 in chronically WHV-infected woodchucks were comparable to those of uninfected woodchucks. In addition, wPD-L1 was expressed in HCC tissue at a lower level than the base line (Fig. 4C).

The MxA expression was a sensitive and specific marker for IFN action in liver. In naïve woodchucks, the measured wMxA expression level was comparable to the one of wPD-L1. The wMxA expression was greatly elevated in livers from woodchucks with acute and chronic WHV infection (Fig. 4C). Thus, wMxA and wPD-L1 were differentially regulated in the liver tissue though both genes were IFN-stimulated genes and responded to IFN-α and -γ in PWHs.

### Generation of specific antibodies to wPD-L1 and -L2

**Figure 5:**
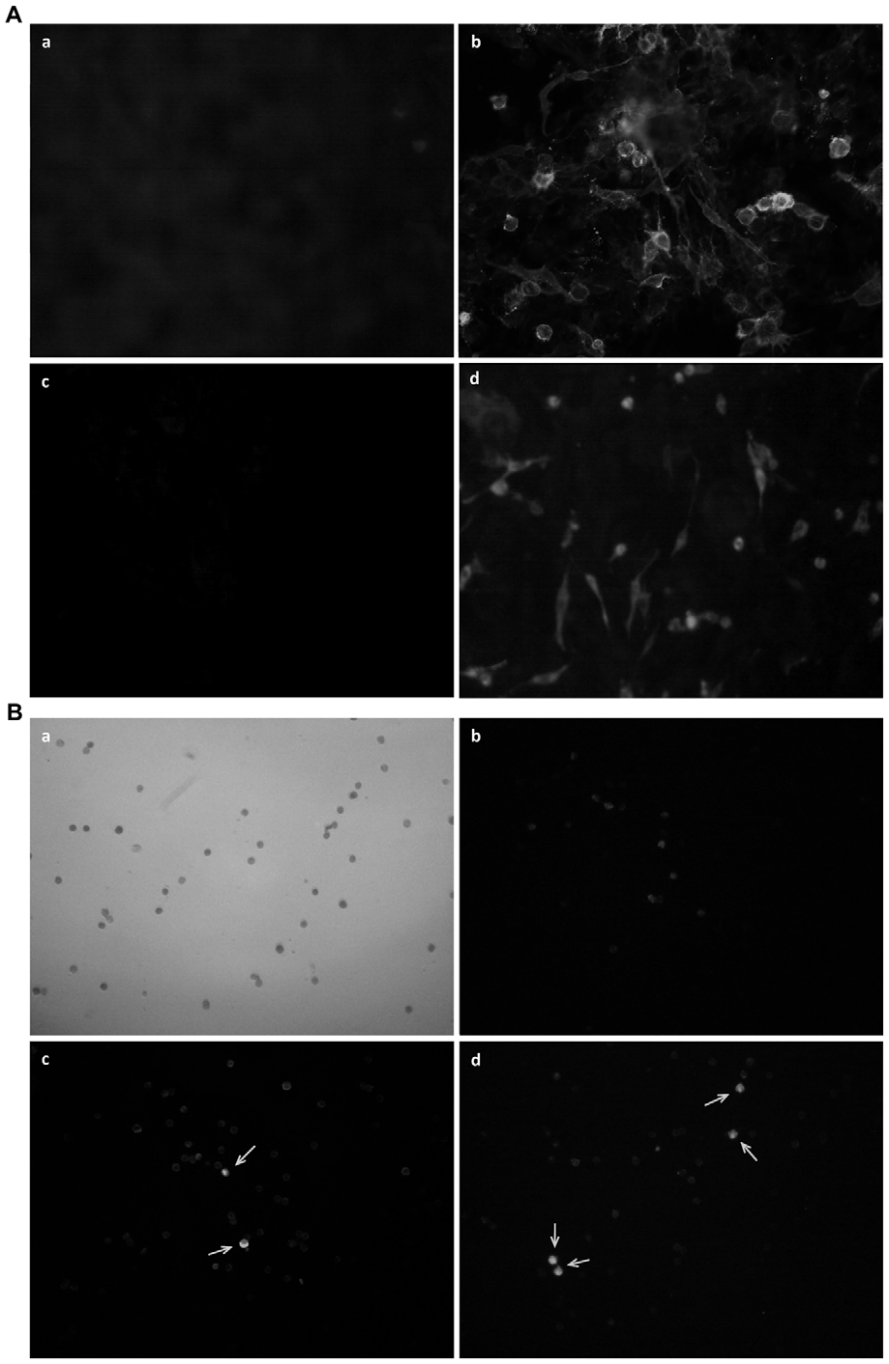
Detection of wPD-L1 and wPD-L2 by specific antisera. BHK cells were transiently transfected with pCI-wL1. or pxf3H-wL2 plasmids. Transfected cells were fixed for IF staining after 48 h. (A) Untransfected cells stained by anti-wPD-L1 (a) or anti-wPD-L2 (c), cells transfected with pCI-wL1 and stained with rabbit anti-wPD-L1 antiserum (b), cells transfected with pxf3H-wL2 and stained with rabbit anti-wPD-L2 antiserum (d). (B) Woodchuck PBMCs in phase contrast (a) and IF staining of woodchuck PBMCs with control antibody (b), anti-wPD-L1 (c), or anti-wPD-L2 (d) antibody. The positively stained cells are indicated by arrows.

Anti-wPD-L1 and -L2 specific antibodies were generated by immunizations of rabbits with recombinant wPD-L1 and -L2 proteins (Text S1 and S2) and tested positively in ELISA and western blotting (Figure S4). Further, BHK cells transiently transfected with pCI-wL1 showed a staining on the cellular membrane in IF staining with rabbit anti-wPD-L1 antibody (Fig. 5A.b). Transfection with pxf3H-wL2 in BHK cells and IF staining with rabbit anti-wPD-L2 resulted in the positive staining of whole cells, as the recombinant proteins is located in the cytoplasma (Fig. 5A.d). Woodchuck PBMC preparations were stained with anti-wPD-L1 and -L2 antibodies. Only a small portion of cells was positively stained at the cytoplasmic membrane (3% and 8% of PBMCs for wPD-L1 and wPD-L2, respectively, Fig. 5B), indicating that wPD-L1 and -L2 were expressed on a part of woodchuck PBMCs, consistent with the previous analysis by flow cytometry.

### Analysis of WHV-specific T cell functions by blockade of wPD-L1 and -L2

Further, we asked whether a blockade of wPD-L1 and -L2 by using specific antibodies may have an influence on T cell functions in acute and chronic WHV infection. A number of commercially available or self-made polyclonal and monoclonal antibodies were tested for in vitro blockage of PD-L1. The rabbit anti-wPD-L1 polyclonal antibody raised in our lab had a stronger blocking effect than other commercially available cross-reactive antibodies. Therefore, IgG fractions prepared from the rabbit polyclonal antibody were used for blockade assay.

Fresh PBMCs were prepared from woodchucks including naïve (n = 7), acutely (n = 8), and chronically WHV-infected (n = 14), and stimulated with WHcAg or WHcAg-derived peptides in the presence or absence of anti-wPD-L1 or anti-wPD-L2 antibodies (Tab. 1). The proliferation of woodchuck PBMCs and degranulation of T cells were determined. The stimulation fold change post blockade was calculated as described and the fold change >2 was regarded as a positive result.

**Figure 6:**
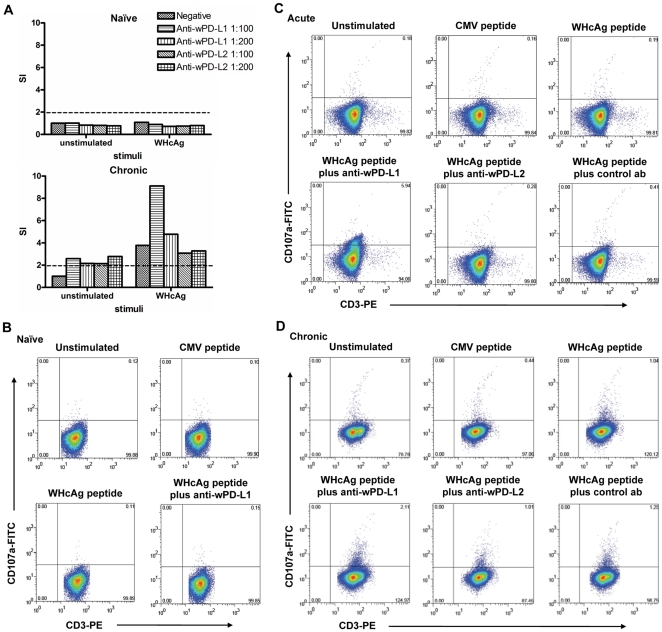
Blockade of the PD-1/PD-Ls pathway by specific antibodies increases specific T cell functions. Woodchuck PBMCs were incubated with anti-wPD-L1, anti-wPD-L2 or anti-wPD-L1 plus anti-wPD-L2 at different concentrations and tested for proliferation and degranulation. (A) Woodchuck PBMCs were stimulated with WHcAg for 5 days. Proliferation of PBMCs was measured by 2-[^3^H] adenine incorporation. (B–D) Woodchuck PBMCs were stimulated with WHcAg derived peptide or control peptide for 2 days. Antigen-specific T cell degranulation was detected by CD107a staining for PBMCs from naïve, acute and chronic WHV infected woodchuck. Control ab, control IgG from normal rabbit serum.

An increase of PBMC proliferation or degranulation after treatment with antibodies to wPD-L1 and -L2 was found with PBMCs from different woodchucks (Table 1, Table S5). Firstly, PBMCs from naïve woodchucks did not show any response to WHV proteins or peptides with or without anti-wPD-L1 and -L2 (Fig. 6A and 6B). The proliferative response of woodchuck PBMCs from woodchucks in the early stage of acute WHV infection (<8 weeks) was not improved by treatment with anti-wPD-L1 and -L2 (Tab. 1, Figure S5 and S6). However, WHcAg-specific CD3+CD4− T cells from 2 of 4 animals showed a stronger degranulation in the presence of anti-wPD-L1 and WHc peptide at week 4 (2- fold) or week 6 (26- fold) post infection without an effect on proliferation, compared to WHcAg-derived peptide only (Fig. 6C), with no effect on proliferation of the cells. In the chronic carrier group, WHcAg-specific CD3+CD4− T cell degranulation in vitro was significantly improved in 5 of 14 woodchucks after the blockade with anti-wPD-L1 (Fig. 6D). In 3 woodchucks, the blockade with either anti-wPD-L1 or -L2 could enhance WHcAg-specific T cell proliferation. However, a blockade with anti-wPD-L2 did not change T cell degranulation in any animals.

**Table 1:** Enhancement of proliferation and degranulation by blockade of wPD-L1 or wPD-L2 with specific antibodies.

		Naïve	Acute (<8w)	Acute (>8w)	Chronic
Total number of woodchucks		7	4	4	14
PD-L1	Proliferation	0	0	0	3
	Degranulation	0	2	0	5*
PD-L2	Proliferation	0	-	-	3
	Degranulation	0	-	-	0

PBMCs from naïve, acutely and chronically infected woodchucks were stimulated with WHcAg or WHcAg-derived peptides in the presence or absence of anti-wPD-L1 or anti-wPD-L2 antibody. The proliferation and degranulation of woodchuck PBMCs were determined. The number of woodchucks which showed an enhanced proliferation or degranulation is given in the table.

*Samples were anlayzed by Pearson chi-square/Fisher's exact test, comparing the effect of control antibody and anti-wPD-L1 antibody (P = 0.015).

## Discussion

In the present work, we examined the woodchuck PD-1/PD-L1 in relation to WHV infection. We could show that both wPD-1 and wPD-L1 expression in woodchuck PBMCs was upregulated during WHV infection though the change of wPD-L1 expression in woodchuck liver tissue was not significant. An enhancement of T cell function was evident in an *in vitro* blockade of the woodchuck PD-1/PD-Ls pathway using antibodies to wPD-L1 and -L2. PBMCs from some chronically as well as acutely WHV-infected woodchucks showed higher proliferative responses and CD107 degranulation activities after the blockade with antibodies to wPD-L1 and -L2.

Recent research indicates that the expression of PD-L1 could be upregulated by different stimuli such as TLR agonists and inflammatory cytokines such as IFNs [22]–[24], as shown for PWHs and woodchuck PBMCs in this study. PWH from the animals with or without WHV infection showed similar reactivity to the stimuli, presumably because hepatocytes reached the same state after culturing overnight in vitro. PBMCs from different animals, which were directly stimulated after purification, showed conflicting response to TLR2/6, 5, 9, and seemed to be influenced by WHV infection. Moreover, PD-1/PD-L1 expression on PBMCs was related to WHV infection. In acute WHV infection, wPD-1 expression on woodchuck PBMCs was induced early during the viremia phase while wPD-L1 upregulation occurred at the late phase of acute infection. Consistent with the human data, both wPD-1 and -L1 expression were elevated in the chronic carriers compared with naive animals [25], [26]. Thus, PD-1/PD-L1 system plays an important role in WHV infected woodchucks.

It was rather surprising, that the wPD-L1 mRNA level was not elevated in the liver tissue from chronically WHV-infected woodchucks, as IFNs were produced by various immune cells in the liver tissue during the hepadnaviral infections and stimulated wMxA expression. Thus, the wPD-L1 expression is differently regulated to that in ISGs and may be only enhanced at high concentrations of IFNs. Another possible explanation is the restricted high expression of PD-L1 in hepatic cells, mainly in liver sinusoidal endothelial cells and Kuppfer cells [27]–[29]. Immunohistochemical staining of PD-L1 revealed that PD-L1 may be upregulated in the liver tissue of chronically HBV-infected patients [12]. Such specific hepatic cell types play an important role in the induction of immune tolerance through the PD-1/PD-L1 system [30], [31]. It is likely that only a small part of hepatic cells expresses high levels of PD-L1 and serves as the major regulator of T cell responses in liver tissue.

The proliferative capacity and functions like cytokine production of specific T cells to viral antigens may be improved by blocking PD-L1 or PD-L2 with specific antibodies *in vitro*. Barber *et al.* showed in the LCMV infection model that a blockade of PD-1/PD-L1 did indeed lead to a restoration of T cell functions and the suppression of viral infection *in vivo* [10]. The *in vivo* blockade using monoclonal antibodies in the Simian immunodeficiency virus-infected macaques results in the rapid expansion of virus-specific CD8+ T cells with a decrease of viral replication. However, several weeks after the blockade, either the T cells or the viral load returned to the previous status [32]. Recently, Fisicaro *et al.* showed that a blockade with anti-PD-L1 promoted the function of HBV-specific T cells derived from chronic HBV patients by affecting the phenotype and function of both peripheral and intrahepatic T cells [13].

In our study, blockade of the wPD-1/PD-L1 pathway could enhance the proliferation and degranulation of woodchuck PBMCs in response to WHcAg in some of the chronic WHV infected woodchucks. A blockade with anti-wPD-L1 antibody enhanced proliferation in two different chronically WHV-infected animals even without WHcAg. Since antigen-presenting cells from chronically WHV-infected animals may already present some unrelated antigens to T-cells, a blockade of PD-1/PD-L1 pathway will generally enhance T-cell proliferation without exogenous proteins. The addition of exogenous WHcAg may preferentially enhance the presentation of that antigen and therefore confer the specificity of stimulation.

However, not every chronic woodchuck was responsive to the blockade, which indicates that the wPD-1/PD-L1 system may be not the only cause for low T cell responses in chronic WHV infection. It is known that the viral specific T cell response was strongly influenced by the viral loads [7], [8]. Recent publications indicated that other inhibitory molecules such as Tim3 and 2B4 were involved in the T cell exhaustion and may be cooperative with PD-1/PD-L1 pathway during chronic viral infections [33], [34]. Thus, a combined approach, for example, with antibodies to such inhibitory molecules and antiviral drugs, would be useful to restore T cell functions in chronically infected individuals. During acute viral infections including HBV infection, PD-1 expression on peripheral blood lymphocytes was often up-regulated [35], [36]. Here we also found the blockade of the wPD1/PD-L1 pathway with anti-wPD-L1 could enhance the specific T cell function for a short time after WHV infection. PBMCs from an earlier or later stage of acute infection showed no response to the antibody blockade. Future experiments *in vivo* will clarify whether a blockade of the PD-1/PD-L1 signalling may break the immune tolerance in chronic HBV infections and have therapeutic effects.

## Supporting Information

Text S1 **Expression of recombinant wPD-L1 and -L2 proteins in E. coli and mammalian cells.**
(DOCX)

Text S2 **Generation of specific antibodies to wPD-L1 and -L2.**
(DOCX)

Figure S1 **A summary of the RT-PCR strategy for amplification of wPD-L1 and -L2.** Primers used to amplify the complete coding sequences of wPD-L1 and -L2 were designed according to the conserved regions among the known PD-L1 and -L2 sequences of other mammalian species. Woodchuck RNAs were extracted from woodchuck liver samples using the TRIZOL reagents and subjected to RT-PCR for amplification of cDNAs of wPD-L1 and -L2. The positions of primers are indicated according to the reference sequences of wPD-L1 (EU306520) and -L2 (EU306521). The secondary structure of wPD-L1 and wPD-L2 proteins was predicted by online analysis and compared with the structure features of PD-L1 and L2 of other mammalian species. Sig, the predicated signal peptide; Ig-V-like, the immunoglobulin V-like domain; Ig-C-like, the immunoglobulin C-like domain; TM, the transmembrane region; Intra, the intracellular domain.
(TIF)

Figure S2 **Flow cytometry analysis of wPD-L1 expression of woodchuck PWHs.** Woodchuck PWHs were treated with TLR ligands, woodchuck IFN-α and -γ for 20 h, detached by trypsin-free cell dissociation buffer, and stained with a commercially available, cross-reactive monoclonal antibody, anti-PD-L1-PE (clone MIH5, ebioscience, USA) and 7aad. For the analysis, hepatocytes were gated and dead cells were excluded as 7aad-positive cells.
(TIF)

Figure S3 **Flow cytometry analysis of wPD-1 and wPD-L1 expression on woodchuck PBMCs.** Woodchuck PBMCs were treated with ligands of TLR3 (poly I∶C, 12.5 µg/ml) and TLR7 (imiquimod, 10 µg/ml) by transfection with lipofectamin 2000 or direct administration of ligands of TLR1/6 (Pam3Cysk4, 2 µg/ml), TLR4 (lipopolysaccharid (LPS), 12.5 µg/ml), and woodchuck IFN-γ (500 U/ml) for 20 h. Two commercially available, cross-reactive monoclonal antibodies, anti-PD-1-FITC (clone J116, ebioscience, USA) and anti-PD-L1-PE (clone MIH5, ebioscience, USA), were used for FACS staining. Woodchuck PBMCs were stained with anti-CD3/anti-CD4/7-amino-actinomycin D (7aad)/anti-PD-1 or anti-CD4/7aad/anti-PD-L1. (A) Fresh woodchuck PBMCs were divided in three populations R1, R2, and R3. R1 was excluded as cell debris and erythrocytes for analysis. R2 contained lymphocytes, as stained with anti-CD3 and anti-CD4. R3 contained CD3− cells with high granularity, representing mixed non-T cell populations. (B) For the analysis of wPD-1 and wPD-L1, woodchuck PBMCs were divided in non-lymphocytes and lymphocytes, further in CD3+CD4− and CD3+CD4+ cells. Dead cells were excluded as 7aad+ cells. (C) Analysis of wPD-L1 expression on woodchuck non-lymphocytes without and after stimulation with TLR ligands. (D) Analysis of wPD-L1 expression on woodchuck lymphocytes without and after stimulation with TLR ligands.
(TIF)

Figure S4 **Detection of recombinant wPD-L1 and -L2 expressed by transient transfection by specific antibodies.** BHK cells were transiently transfected with pxf3H-wL1 or pxf3H-wL2 plasmids. Transfected cells were fixed for IF staining after 48 h. Western blotting of transfected cells stained by control rabbit sera, anti-HA monoclonal antibody, anti-wPD-L1 or anti-wPD-L2 antisera. In SDS-PAGE and western blotting analysis, cells transfected with pxf3H-wL1 and pxf3H-wL2 expressed specific protein bands at the molecular weight of about 30 kD that were detected by anti-HA and antisera to wPD-L1 and -L2, respectively, corresponding to HA-wPD-L1 and -L2 with HA-tag MYP YDV PDY ANS PYP YDV PDY AEF. No band was recognized when cells were transfected with an empty vector.
(TIF)

Figure S5 **Blocking the PD-1/PDLs pathway in vitro enhanced the antigen specific proliferation of woodchuck PBMCs.** Woodchuck PBMCs were stimulated with WHcAg or ConA for 5 days with or without blockage with anti-PDL1, anti-PDL2, or anti-PDL1 plus anti-PDL2 at different concentrations. Proliferation of woodchuck PBMCs was measured by 2-[3H] adenine incorporation. In naïve animals and some of the chronically infected animals, the blockage with antibodies to wPD-L1 and –L2 had no effect on lymphoproliferation (A) while an enhancement of the antigen specific proliferation at different level was measured with PBMCs from some other chronic carriers (B).
(TIF)

Figure S6 **Blocking the PD-1/PDLs pathway in vitro enhanced the antigen specific CD107a degranulation of woodchuck PBMCs.** Woodchuck PBMCs were stimulated with WHcAg derived peptide or control peptide for 2 days with or without blockage with anti-PDL1, anti-PDL2, or unrelated antibody preparations. Antigen-specific CD107a degranulation was detected by CD107a staining for PBMCs from naïve, acutely and chronically WHV-infected woodchucks. In naïve animals and some of the chronically infected animals, the blockage with antibodies to wPD-L1 and –L2 had no effect on CD107a degranulation (A) while an enhancement of the antigen-specific CD107a degranulation at different levels was measured with PBMCs from some other chronic carriers (B).
(TIF)

Table S1 **The primers used for RT-PCR amplification of wPDL1 and wPDL2.**
(DOCX)

Table S2 **Homology of wPD-L1 and wPD-L2 to the counterparts of other mammalian species on the nucleotide and amino-acid level.**
(DOCX)

Table S3 **Raw data of wPD-L1 expression in PWH after TLR stimulation.**
(DOCX)

Table S4 **Raw data of wPD-L1 expression in PBMCs after TLR stimulation.**
(DOCX)

Table S5 **Blockage with antibodies to wPD-L1 and -L2 enhances the antigen specific CD107a degranulation in some woodchucks.**
(DOCX)
