# Supplementary material for: The Expression of PD-1 Ligands and Their Involvement in Regulation of T Cell Functions in Acute and Chronic Woodchuck Hepatitis Virus Infection
Source: PLoS One. 2011 Oct 14;6(10):e26196. doi: 10.1371/journal.pone.0026196 (PMC3194835; doi:10.1371/journal.pone.0026196)
Supplement: Text S1 — Expression of recombinant wPD-L1 and -L2 proteins in E. coli and mammalian cells. (DOCX) [file pone.0026196.s001.docx]

**Text S1. Expression of recombinant wPD-L1 and -L2 proteins in E. coli and mammalian cells**

To construct the prokaryotic expression vectors, the coding sequences nucleotide (nt) 38-698 and nt 39-638 for the extracellular region in the putative mature wPD-L1 and -L2 proteins were amplified with specific primer pairs wPDL1-P5/P6 and wPDL2-P5/P6 respectively, and ligated into the vector pET-30a (Merck, Germany) at the EcoRI and SalI sites. The recombinant proteins His-wPD-L1 and -L2 were expressed in the *E. coli* strain BL21 (DE plus) and purified with HisTrap Kit (Amersham, Braunschweig, Germany) under denaturation conditions according to manufacturer's instructions.

Two mammalian expression vectors for wPD-L1 and -L2 with a HA-tag were constructed on the basis of pxf3H (kindly provided by Dr. Xinhua Feng). The fragments were generated by PCR using the specific primer pairs wPDL1-P5/P7 and wPDL2-P5/P7, respectively. One mammalian expression vector for wPD-L1 with the complete coding region was constructed on the basis of pCI-neo Mammalian Expression Vector (Promega, USA) using the primers wPDL1-P8/P9. These procedures resulted into the mammalian expression vectors pxf3H-wL1, pxf3H-wL2, and pCI-wL1.
