# Supplementary material for: The Expression of PD-1 Ligands and Their Involvement in Regulation of T Cell Functions in Acute and Chronic Woodchuck Hepatitis Virus Infection
Source: PLoS One. 2011 Oct 14;6(10):e26196. doi: 10.1371/journal.pone.0026196 (PMC3194835; doi:10.1371/journal.pone.0026196)
Supplement: Text S2 — Generation of specific antibodies to wPD-L1 and -L2. (DOCX) [file pone.0026196.s002.docx]

**Text S2. Generation of specific antibodies to wPD-L1 and -L2**

Rabbits were immunized by subcutaneous immunization with 200 μg of purified recombinant His-wPD-L1 and -L2 proteins in incomplete Freund's adjuvant at 4 week intervals, respectively. The animals were sacrificed on day 14 post the 3^rd^ immunization and the blood were obtained.

The titers of anti-wPD-L1 and anti-wPD-L2 were detected by enzyme-linked immunosorbent assay (ELISA) using the purified His-wPD-L1 and -L2 proteins, and then calculated by extrapolation of ELISA values of serially diluted samples. Corresponding to the reciprocal values of the highest dilutions that were regarded as positive, the titers of anti-wPD-L1 and -L2 were >1:500,000 and >1:1,000,000, respectively. No cross-reactivity between anti-wPD-L1 and –L2 was detected in ELISA.

Cells transfected with pXF-wPD-L1 and pXF-wPD-L2 were harvested and subjected to SDS-PAGE and western blotting with anti-HA, antis-wPD-L1 or -L2, respectively. The specific bands at the molecular weight of 30 kD were recognized for both wPD-L1 and wPD-L2, corresponding to the predicted sizes. (Supplementary Fig.4). No band was recognized when cells were transfected with an empty vector. These results indicated the specificity of antis-wPD-L1 and wPD-L2.
