# Supplementary figures and images for: The Expression of PD-1 Ligands and Their Involvement in Regulation of T Cell Functions in Acute and Chronic Woodchuck Hepatitis Virus Infection
Source: PLoS One. 2011 Oct 14;6(10):e26196. doi: 10.1371/journal.pone.0026196 (PMC3194835; doi:10.1371/journal.pone.0026196)

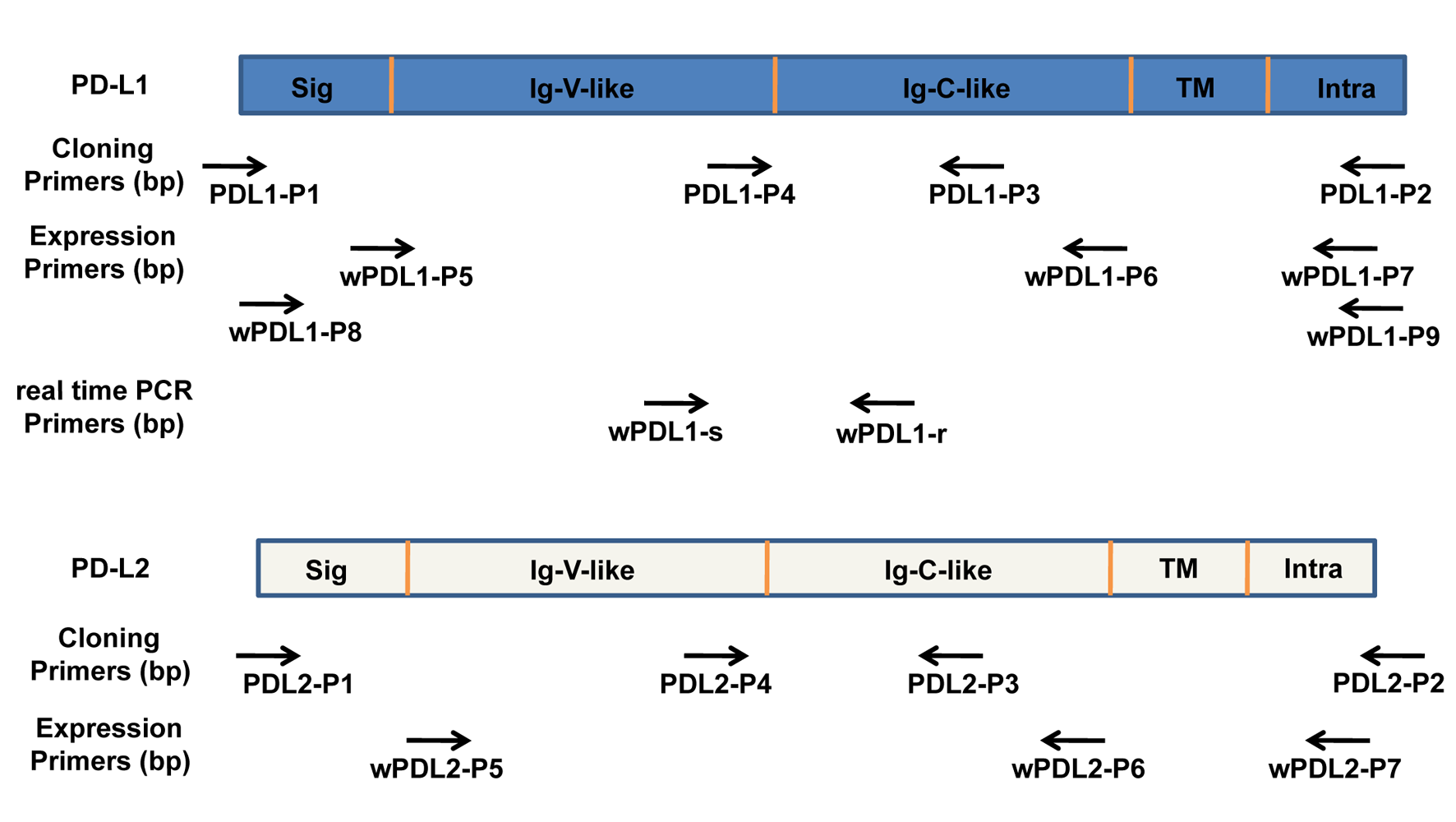

Supplement: Figure S1 — A summary of the RT-PCR strategy for amplification of wPD-L1 and -L2. Primers used to amplify the complete coding sequences of wPD-L1 and -L2 were designed according to the conserved regions among the known PD-L1 and -L2 sequences of other mammalian species. Woodchuck RNAs were extracted from woodchuck liver samples using the TRIZOL reagents and subjected to RT-PCR for amplification of cDNAs of wPD-L1 and -L2. The positions of primers are indicated according to the reference sequences of wPD-L1 (EU306520) and -L2 (EU306521). The secondary structure of wPD-L1 and wPD-L2 proteins was predicted by online analysis and compared with the structure features of PD-L1 and L2 of other mammalian species. Sig, the predicated signal peptide; Ig-V-like, the immunoglobulin V-like domain; Ig-C-like, the immunoglobulin C-like domain; TM, the transmembrane region; Intra, the intracellular domain. (TIF) [file pone.0026196.s003.tif]

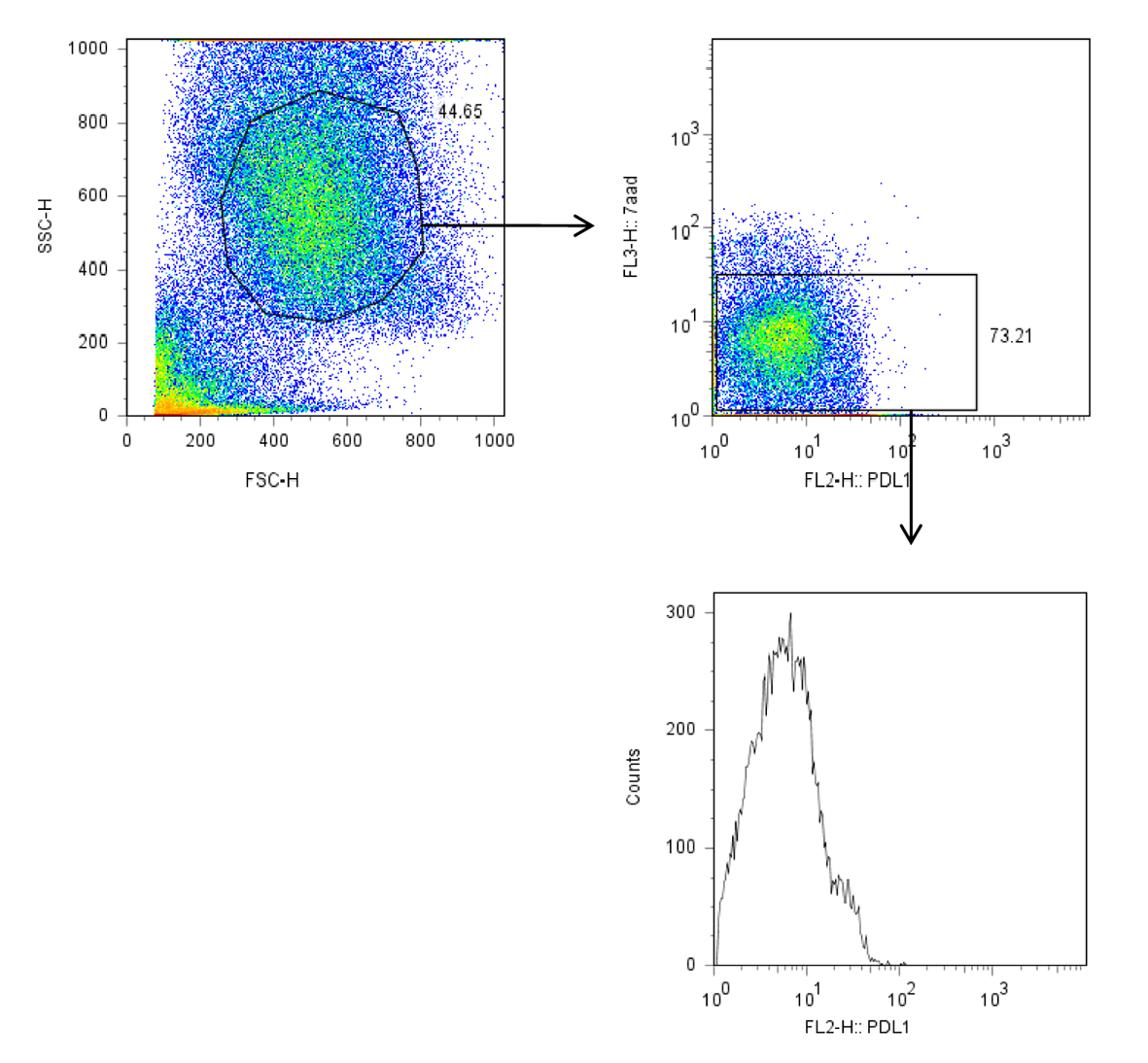

Supplement: Figure S2 — Flow cytometry analysis of wPD-L1 expression of woodchuck PWHs. Woodchuck PWHs were treated with TLR ligands, woodchuck IFN-α and -γ for 20 h, detached by trypsin-free cell dissociation buffer, and stained with a commercially available, cross-reactive monoclonal antibody, anti-PD-L1-PE (clone MIH5, ebioscience, USA) and 7aad. For the analysis, hepatocytes were gated and dead cells were excluded as 7aad-positive cells. (TIF) [file pone.0026196.s004.tif]

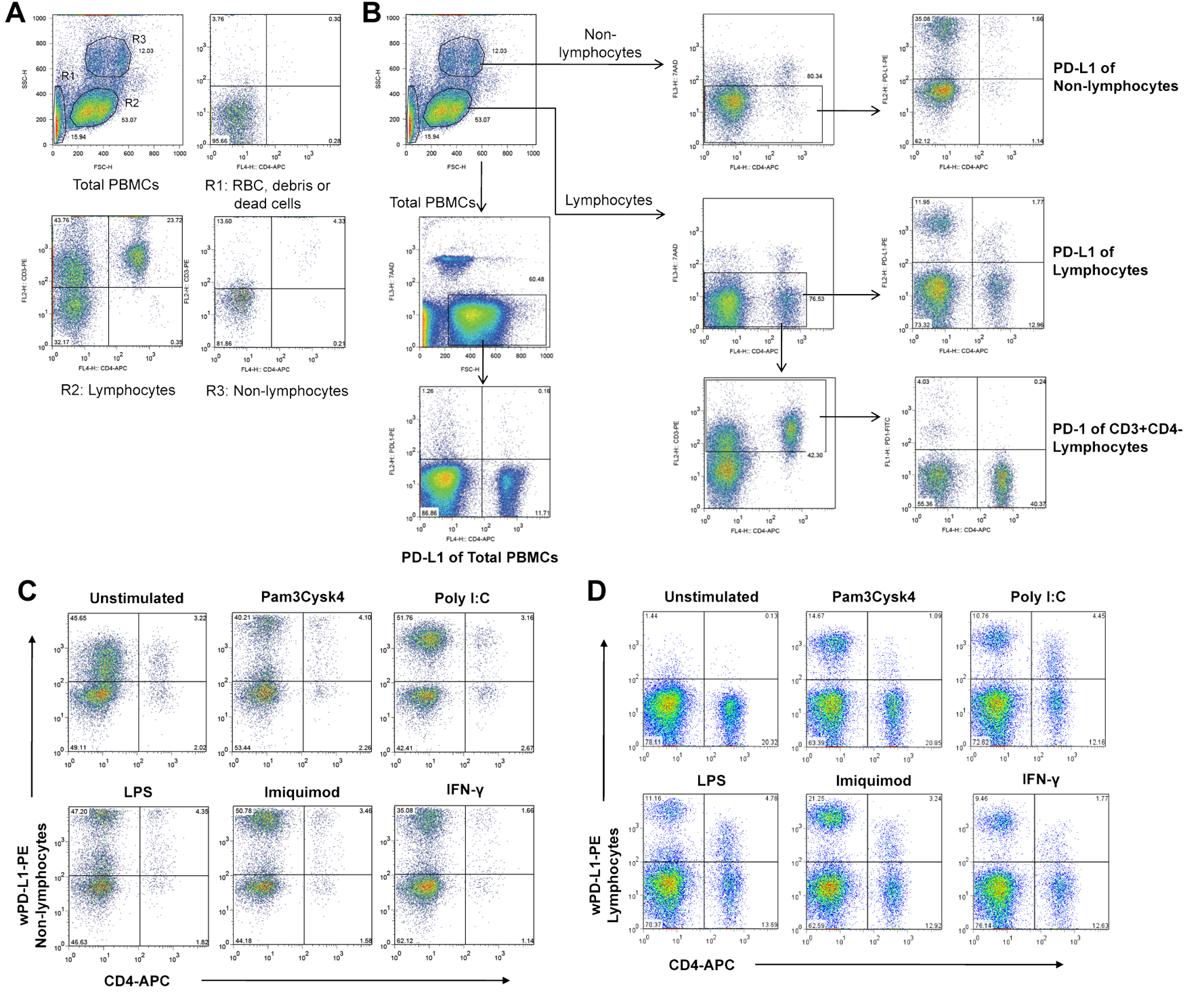

Supplement: Figure S3 — Flow cytometry analysis of wPD-1 and wPD-L1 expression on woodchuck PBMCs. Woodchuck PBMCs were treated with ligands of TLR3 (poly I∶C, 12.5 µg/ml) and TLR7 (imiquimod, 10 µg/ml) by transfection with lipofectamin 2000 or direct administration of ligands of TLR1/6 (Pam3Cysk4, 2 µg/ml), TLR4 (lipopolysaccharid (LPS), 12.5 µg/ml), and woodchuck IFN-γ (500 U/ml) for 20 h. Two commercially available, cross-reactive monoclonal antibodies, anti-PD-1-FITC (clone J116, ebioscience, USA) and anti-PD-L1-PE (clone MIH5, ebioscience, USA), were used for FACS staining. Woodchuck PBMCs were stained with anti-CD3/anti-CD4/7-amino-actinomycin D (7aad)/anti-PD-1 or anti-CD4/7aad/anti-PD-L1. (A) Fresh woodchuck PBMCs were divided in three populations R1, R2, and R3. R1 was excluded as cell debris and erythrocytes for analysis. R2 contained lymphocytes, as stained with anti-CD3 and anti-CD4. R3 contained CD3− cells with high granularity, representing mixed non-T cell populations. (B) For the analysis of wPD-1 and wPD-L1, woodchuck PBMCs were divided in non-lymphocytes and lymphocytes, further in CD3+CD4− and CD3+CD4+ cells. Dead cells were excluded as 7aad+ cells. (C) Analysis of wPD-L1 expression on woodchuck non-lymphocytes without and after stimulation with TLR ligands. (D) Analysis of wPD-L1 expression on woodchuck lymphocytes without and after stimulation with TLR ligands. (TIF) [file pone.0026196.s005.tif]

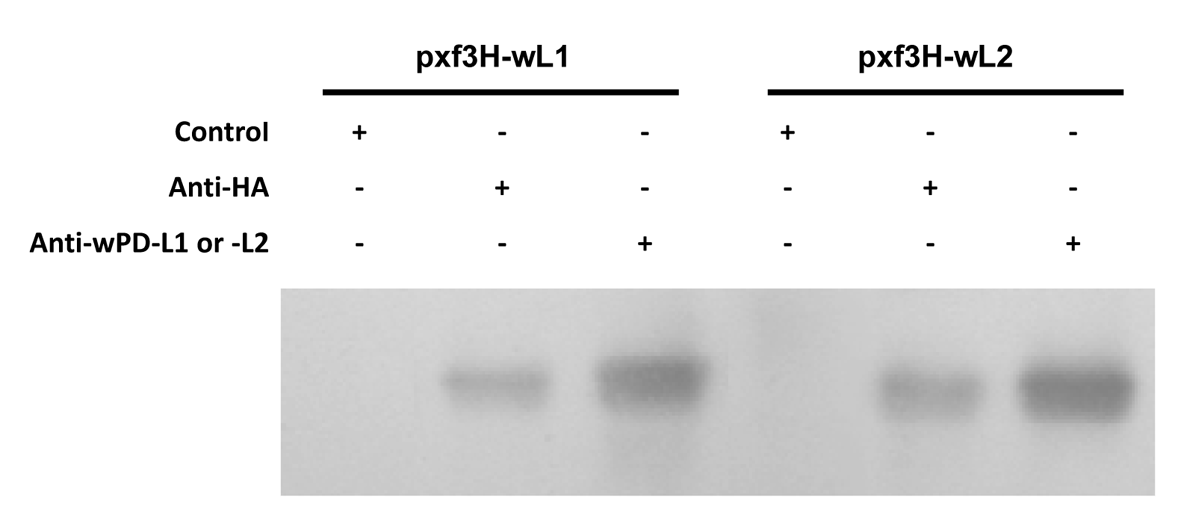

Supplement: Figure S4 — Detection of recombinant wPD-L1 and -L2 expressed by transient transfection by specific antibodies. BHK cells were transiently transfected with pxf3H-wL1 or pxf3H-wL2 plasmids. Transfected cells were fixed for IF staining after 48 h. Western blotting of transfected cells stained by control rabbit sera, anti-HA monoclonal antibody, anti-wPD-L1 or anti-wPD-L2 antisera. In SDS-PAGE and western blotting analysis, cells transfected with pxf3H-wL1 and pxf3H-wL2 expressed specific protein bands at the molecular weight of about 30 kD that were detected by anti-HA and antisera to wPD-L1 and -L2, respectively, corresponding to HA-wPD-L1 and -L2 with HA-tag MYP YDV PDY ANS PYP YDV PDY AEF. No band was recognized when cells were transfected with an empty vector. (TIF) [file pone.0026196.s006.tif]

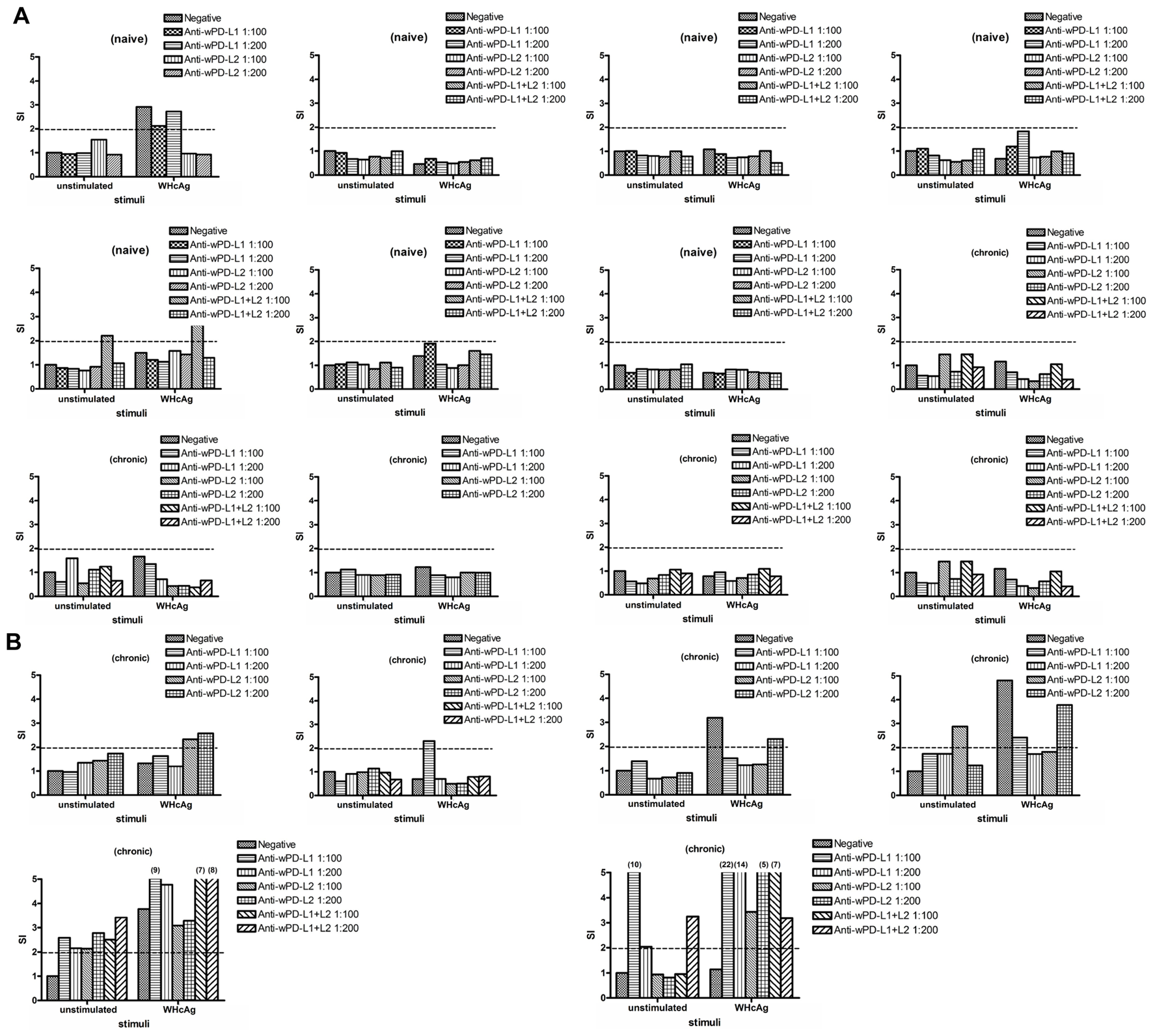

Supplement: Figure S5 — Blocking the PD-1/PDLs pathway in vitro enhanced the antigen specific proliferation of woodchuck PBMCs. Woodchuck PBMCs were stimulated with WHcAg or ConA for 5 days with or without blockage with anti-PDL1, anti-PDL2, or anti-PDL1 plus anti-PDL2 at different concentrations. Proliferation of woodchuck PBMCs was measured by 2-[3H] adenine incorporation. In naïve animals and some of the chronically infected animals, the blockage with antibodies to wPD-L1 and –L2 had no effect on lymphoproliferation (A) while an enhancement of the antigen specific proliferation at different level was measured with PBMCs from some other chronic carriers (B). (TIF) [file pone.0026196.s007.tif]

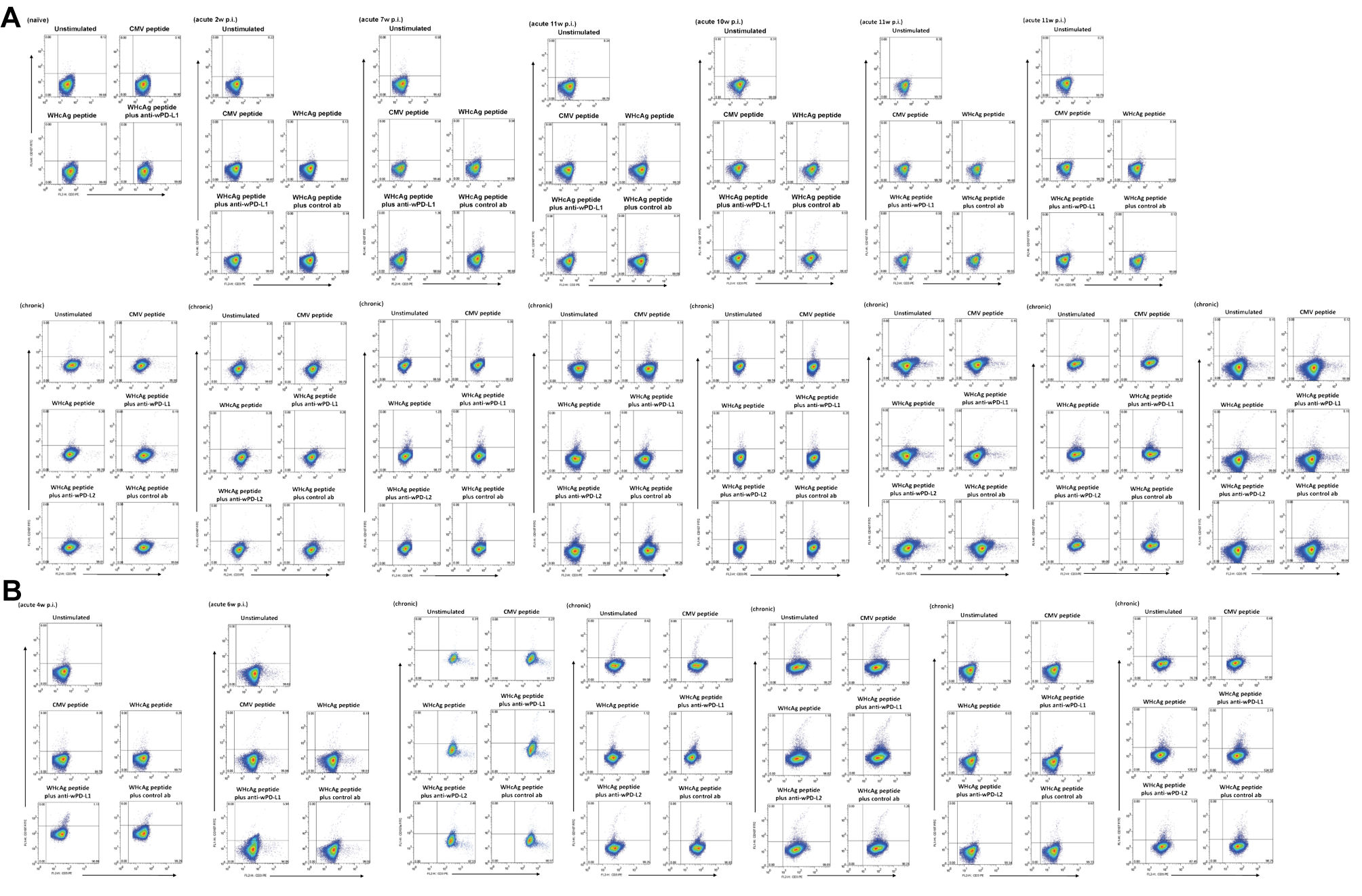

Supplement: Figure S6 — Blocking the PD-1/PDLs pathway in vitro enhanced the antigen specific CD107a degranulation of woodchuck PBMCs. Woodchuck PBMCs were stimulated with WHcAg derived peptide or control peptide for 2 days with or without blockage with anti-PDL1, anti-PDL2, or unrelated antibody preparations. Antigen-specific CD107a degranulation was detected by CD107a staining for PBMCs from naïve, acutely and chronically WHV-infected woodchucks. In naïve animals and some of the chronically infected animals, the blockage with antibodies to wPD-L1 and –L2 had no effect on CD107a degranulation (A) while an enhancement of the antigen-specific CD107a degranulation at different levels was measured with PBMCs from some other chronic carriers (B). (TIF) [file pone.0026196.s008.tif]
