# Supplementary material for: The Expression of PD-1 Ligands and Their Involvement in Regulation of T Cell Functions in Acute and Chronic Woodchuck Hepatitis Virus Infection
Source: PLoS One. 2011 Oct 14;6(10):e26196. doi: 10.1371/journal.pone.0026196 (PMC3194835; doi:10.1371/journal.pone.0026196)
Supplement: Table S1 — The primers used for RT-PCR amplification of wPDL1 and wPDL2. (DOCX) [file pone.0026196.s009.docx]

**Table S1. The primers used for RT-PCR amplification of wPDL1 and wPDL2.**

| Name |  | Polarity | |  | | Sequence |  | position | |
| --- | --- | --- | --- | --- | --- | --- | --- | --- | --- |
| Primers for RT-PCR and cloning* | | | | | | | | | |
| PDL1-P1 | | sense | | | 5'-CAGGGCATTCCAGAAAGATGAGGAT-3' | | | | -17* |
| PDL1-P2 | | antisense | | | 5'-TTACGTCTCCTCCAAATGTGTATC-3' | | | | 864* |
| PDL1-P4 | | sense (nested ) | | | 5'-TGGTGCCGACTACAAGC-3' | | | | 357* |
| PDL1-P3 | | antisense (nested ) | | | 5'-ATTCTCAGTGTGCTGGTCAC-3' | | | | 593* |
| PDL2-P1 | | sense | | | 5'-ATGATCTTCCTCCTGCTAAT-3 | | | | 1* |
| PDL2-P2 | | Antisense | | | 5'-TCAGATAGCACTGTTCACTTCCCTC-3' | | | | 822* |
| PDL2-P4 | | sense (nested ) | | | 5'-ATGGGGTCGCCTGGGACTAC-3 | | | | 317* |
| PDL2-P3 | | Antisense (nested ) | | | 5'-GAACACTGGTGACCTGGTAG-3' | | | | 538* |
| Primers for subcloning and construction of expression vectors** | | | | | | | | | |
| wPDL1-P5 | | sense | 5'-CCGGAATTCCTGAACGCCTTTAGTATC-3' | | | | | | 38 |
| wPDL1-P6 | | antisense | 5'-ACGCGTCGACGAGTCCTTTGAAGTAAG-3' | | | | | | 698 |
| wPDL1-P7 | | antisense | 5'-ACGCGTCGACTTATATGCCACCATTCTCCAC-3' | | | | | | 804 |
| wPDL1-P8 | | sense | 5-CCG GAA TTCATGAGGATCTTTAATGTCTT-3 | | | | | | 2 |
| wPDL1-P9 | | antisense | 5-ACGC GTCGACTTACGTCTCCTCCAAATGTGTATC -3 | | | | | | 864* |
| wPDL2-P5 | | sense | 5'-CCGGAATTCTTATTCACAGTGACAGTCC-3' | | | | | | 39 |
| wPDL2-P6 | | antisense | 5'-ACGCGTCGACTTAAGTAGGGATGTTGGGTTTC-3' | | | | | | 638 |
| wPDL2-P7 | | antisense | 5'-ACGCGTCGACTCAGATAGCACTGTTCACTTCCCTC-3' | | | | | | 822* |
| Real time-PCR primers*** | | | | | | | | | |
| wPDL1-s | | sense | 5'-GGATGCTGGGGTTTACTGCT-3' | | | | | | 313 |
| wPDL1-r | | antisense | 5'-TCTGGGATTTGGTGATGGTG-3' | | | | | | 545 |
| wMxA-s | | sense | 5'-GGAGGGAGGAGAAGAGGAAA-3' | | | | | | 66 |
| wMxA-r | | antisense | 5'-CTGGAGATGCGGTTGTGAG-3' | | | | | | 189 |
| wβ-actin-s | | sense | 5'-TGGAATCCTGTGGCATCCATGAAAC-3' | | | | | | 1 |
| wβ-actin-r | | antisense | 5'-TAAAACGCAGCTCAGTAACAGTCCG-3' | | | | | | 346 |

*. The primers of PD-L1 and -L2 were designed according to human PD-L1 or PD-L2 sequences AY254342 and AF344424.

** . The positions of primers are indicated according to the reference sequences of wPD-L1 (EU306520) and -L2 (EU306521). Two primers indicated with * were given according to human sequences.

***. The positions of the primers for real time RT-PCR were given according to the reference sequences, wPD-L1 (EU306520), wMxA (EU503128), and wß-actin (AY170121).
