# Supplementary material for: The Expression of PD-1 Ligands and Their Involvement in Regulation of T Cell Functions in Acute and Chronic Woodchuck Hepatitis Virus Infection
Source: PLoS One. 2011 Oct 14;6(10):e26196. doi: 10.1371/journal.pone.0026196 (PMC3194835; doi:10.1371/journal.pone.0026196)
Supplement: Table S2 — Homology of wPD-L1 and wPD-L2 to the counterparts of other mammalian species on the nucleotide and amino-acid level. (DOCX) [file pone.0026196.s010.docx]

**Table S2. Homology of wPD-L1 and wPD-L2 to the counterparts of other mammalian species on the nucleotide and amino-acid level.**

**A. Homology of PD-L1**

| **Species** | Woodchuck | Human | Porcine | Mouse | Cattle |
| --- | --- | --- | --- | --- | --- |
| Woodchuck | 100 | - | - | - | - |
| Human | 84.9 (75.9) | 100 | - | - | - |
| Porcine | 82.1 (71.9) | 85.4 (74.8) | 100 | - | - |
| Mouse | 75.3 (66.2) | 76.7 (69.9) | 74.5 (67.2) | 100 | - |
| Cattle | 80.9 (70.4) | 83.7 (73.6) | 88.4 (82.9) | 73.8 (65.7) | 100 |

**B. Homology of PD-L2**

| **Species** | Woodchuck | Human | Monkey | Porcine | Mouse |
| --- | --- | --- | --- | --- | --- |
| Woodchuck | 100 | - | - | - | - |
| Human | 83.8 (72.9) | 100 | - | - | - |
| Monkey | 83.2 (72.5) | 97.7 (96.0) | 100 | - | - |
| Porcine | 80.3 (68.8) | 81.9 (69.9) | 81.9 (71.3) | 100 | - |
| Mouse | 78.0 (70.9) | 77.6 (69.6) | 76.9 (68.4) | 74.4 (65.4) | 100 |

The following sequences were used for comparison: human PD-L1 (AY254342), woodchuck PD-L1 (EU306520), porcine PD-L1 (AY837780), mouse PD-L1 (NM_021893), cattle PD-L1 (AB510902), human PD-L2 (AF344424), woodchuck PD-L2 (EU306521), monkey PD-L2 (NM_001083599), porcine PD-L2 (NM_001025220), mouse PD-L2 (NM_021396). The numbers outside of brackets indicate the similarities at nt level in percentage; the numbers inside of brackets indicate the similarities at the aa level in percentage.
