# Supplementary material for: The Expression of PD-1 Ligands and Their Involvement in Regulation of T Cell Functions in Acute and Chronic Woodchuck Hepatitis Virus Infection
Source: PLoS One. 2011 Oct 14;6(10):e26196. doi: 10.1371/journal.pone.0026196 (PMC3194835; doi:10.1371/journal.pone.0026196)
Supplement: Table S3 — Raw data of wPD-L1 expression in PWH after TLR stimulation. (DOCX) [file pone.0026196.s011.docx]

**Table S3. Raw data of wPD-L1 expression in PWH after TLR stimulation**

| Stimuli |  | | Fold change | |  | |  | |
| --- | --- | --- | --- | --- | --- | --- | --- | --- |
|  | Woodchuck 1 | | Woodchuck 2 | | Woodchuck 3 | | Woodchuck 4 | |
| Control | 1.1 | 0.7 | 1.3 | 0.7 | 0.9 | 1.3 | 1.1 | 0.9 |
| Pam3Cysk4 | n.d | n.d | 2.9 | 2.4 | 1.6 | 1.4 | 1.1 | 0.8 |
| Pam2Cysk4 | n.d | n.d | 2.6 | 1.8 | 2.0 | 1.5 | 1.3 | 1.0 |
| Poly I:C | 29.2 | 26.0 | 17.3 | 18.3 | 11.8 | 8.6 | 2.7 | 4.2 |
| LPS | 6.4 | 7.0 | 6.3 | 6.5 | 2.2 | 2.5 | 1.6 | 1.9 |
| Flagellin | n.d | n.d | 0.9 | 0.8 | 0.7 | 0.9 | 0.8 | 0.8 |
| Imiquimod | 1.0 | 1.0 | 9.1 | 7.7 | 6.8 | 5.8 | 0.8 | 0.7 |
| CPG ODN | n.d | n.d | 0.9 | 0.9 | 0.8 | 0.9 | 0.9 | 0.9 |
| IFN-a | 3.5 | 2.6 | 4.0 | 3.8 | 2.9 | 2.8 | 6.1 | 7.7 |
| IFN-γ | 3.5 | 3.2 | 3.9 | 5.3 | 2.8 | 3.8 | 8.1 | 6.3 |

Four woodchucks were taken for PWH preparation. In each PWH preparations, cells were treated with TLR ligands in duplicate. The copy numbers of wPD-L1 transcripts in PWH were determined by real time RT-PCR and normalized against beta-actin. The fold change of wPD-L1 expression was calculated with the average wPD-L1 expression levels in stimulated PWHs divided the average of that in untreated PWHs.
