# Supplementary material for: The Expression of PD-1 Ligands and Their Involvement in Regulation of T Cell Functions in Acute and Chronic Woodchuck Hepatitis Virus Infection
Source: PLoS One. 2011 Oct 14;6(10):e26196. doi: 10.1371/journal.pone.0026196 (PMC3194835; doi:10.1371/journal.pone.0026196)
Supplement: Table S4 — Raw data of wPD-L1 expression in PBMCs after TLR stimulation. (DOCX) [file pone.0026196.s012.docx]

**Table S4. Raw data of wPD-L1 expression in PBMCs after TLR stimulation**

| Stimuli |  | |  | | Fold change | |  | |  | |
| --- | --- | --- | --- | --- | --- | --- | --- | --- | --- | --- |
|  | Naive 1 | | Naive 2 | | Chronic 3 | | Chronic 4 | | Chronic 5 | |
| Control | 1.1 | 0.9 | 1.0 | 1.1 | 0.8 | 1.2 | 1.1 | 0.9 | 1.0 | 1.0 |
| Pam3Cysk4 | 3.2 | 3.7 | 2.8 | 3.0 | 2.5 | 2.6 | 2.9 | 2.7 | 3.2 | 3.0 |
| Pam2Cysk4 | 3.5 | 2.8 | 3.6 | 4.5 | 5.1 | 3.1 | 0.8 | 1.2 | 1.4 | 1.0 |
| Poly I:C | 1.5 | 4.1 | 3.3 | 2.0 | 7.1 | 4.0 | 1.3 | 3.3 | 1.5 | 1.0 |
| LPS | 8.5 | 6.9 | 7.9 | 7.4 | 10.0 | 7.4 | 7.5 | 6.8 | 6.1 | 5.8 |
| Flagellin | 1.1 | 1.0 | 0.9 | 1.1 | 1.2 | 1.3 | 1.6 | 5.9 | 3.9 | 4.7 |
| Imquimod | 12.0 | 9.6 | 10.5 | 4.4 | 10.0 | 12.3 | 3.3 | 2.4 | 2.0 | 1.8 |
| CPG ODN | 1.2 | 1.1 | 1.0 | 0.9 | 2.3 | 2.1 | 1.2 | 2.9 | 0.7 | 0.8 |
| IFN-γ | 3.0 | 2.9 | 2.9 | 3.1 | 3.1 | 3.0 | 3.9 | 3.1 | 3.0 | 3.2 |

Two naïve and 3 chronic woodchucks were taken for the experiments. In each PBMC preparations, cells were treated with TLR ligands in duplicate. The copy numbers of wPD-L1 transcripts in PBMCs were determined by real time RT-PCR and normalized against beta-actin. The fold change of wPD-L1 expression was calculated with the average wPD-L1 expression levels in stimulated PBMCs divided the average of that in untreated PBMCs.
