# Supplementary material for: The Expression of PD-1 Ligands and Their Involvement in Regulation of T Cell Functions in Acute and Chronic Woodchuck Hepatitis Virus Infection
Source: PLoS One. 2011 Oct 14;6(10):e26196. doi: 10.1371/journal.pone.0026196 (PMC3194835; doi:10.1371/journal.pone.0026196)
Supplement: Table S5 — Blockage with antibodies to wPD-L1 and -L2 enhances the antigen specific CD107a degranulation in some woodchucks. (DOCX) [file pone.0026196.s013.docx]

**Table S5. Blockage with antibodies to wPD-L1 and -L2 enhances the antigen specific CD107a degranulation in some woodchucks.**

| WHcAg-peptide | +Medium | +Anti-wPDL1 | +Anti-wPDL2 | +Control ab |
| --- | --- | --- | --- | --- |
| chronic | 2.71% | 4.86% | 2.49% | 1.43% |
| chronic | 1.12% | 2.06% | 0.75% | 1.40% |
| chronic | 1/18% | 1.94% | 0.99% | 1.26% |
| chronic | 0.63% | 1.83% | 0.46% | 0.67% |
| chronic | 1.04% | 2.11% | 1.01% | 1.25% |
| acute | 0.19% | 5.94% | 0.20% | 0.41% |
| acute | 0.29% | 1.11% | - | 0.71% |

All cells were stimulated with WHcAg-derived peptide aa96-110 with the indicating antibodies for 3 days. The percentage of CD107+ T cells in CD3+CD4- population is given. The tests were performed with medium control, antibodies to wPD-L1 and -L2, and control antibodies from unimmunized rabbits.
